# Complete genome sequence of an enterotoxigenic *Bacteroides fragilis* strain 86-5443-2-2 from a piglet

**DOI:** 10.1128/mra.00409-24

**Published:** 2025-02-24

**Authors:** Eunbi Lee, Jinki Yeom

**Affiliations:** 1 Department of Biomedical Science, College of Medicine, Seoul National University, Seoul, South Korea; 2 Cancer Research Institute, Seoul National University, Seoul, South Korea; 3 Department of Microbiology and Immunology, College of Medicine, Seoul National University, Seoul, South Korea; University of Maryland School of Medicine, Baltimore, Maryland, USA

**Keywords:** gut, toxin, inflammatory bowel disease, colorectal cancer

## Abstract

This study presents the genome sequence of *Bacteroides fragilis* strain 86-5443-2-2, isolated from a piglet. The genome of this strain forms a single contig spanning 5.4 million base pairs with a 43.5% GC content and 4,480 genes. Notably, this bacterium possesses two copies of a pathogenic island capable of producing enterotoxins.

## ANNOUNCEMENT

The enterotoxigenic *Bacteroides fragilis* (ETBF) strain 86-5443-2-2, isolated from a piglet’s stool in 1996 by Dr. C. Sears’s group (1), is known for its high virulence due to substantial toxin production (2). Prior to this research, the genome of this strain remained at contig level (GenBank ID: LIDS00000000). Our study marks the complete genome sequencing of the ETBF strain 86-5443-2-2.

ETBF strain 86-5443-2-2, obtained as a gift from Dr. C. Sears (1), was stored at −80°C in 20% glycerol stock in our laboratory. For preparation sample, strain was incubated in 30 mL BHI medium (Difco, 237500) supplemented with 5 mg/mL yeast extract, 5 µg/mL hemin, 0.5 µg/mL vitamin K, 200 µg/mL gentamicin, and 0.5 mg/mL L-cysteine in an anaerobic chamber (Whitley DG250, 10% H_2_, 10% CO_2_, and 80% N_2_) at 37°C for 48 hours (3). Genomic DNA was isolated with QIAamp DNA Minikit (Qiagen) following the manufacturer’s protocol. For PacBio sequencing library preparation, 3 µg of DNA was used for 12 kb library preparation. For DNA with a size range of less than 17 kb, FemtoPulse (Agilent) was used to determine the actual size distribution. DNA was sheared with Megaruptor3 (Diagenode) and purified using AMPure PB magnetic beads (PacBio) if the apparent size exceeded 40 kb. A total of 10 µL library was prepared using PacBio SMRTbell Express Template Prep Kit 2.0 (PacBio, PN 100-938-900). For Illumina sequencing library preparation, the TruSeq Nano DNA Kit (Illumina, cat #FC-121-9010DOC) was used following the TruSeq Nano DNA Sample Preparation Guide (Part #15041110 Rev.D). Illumina sequencing was conducted using HiseqXten with a 150 bp read length. The same genomic DNA was used for both PacBio Sequel and Illumina platform sequencing (Table 1).

**TABLE 1:** Sequencing data and genomic features of ETBF strain 86-5443-2-2

Sequencing data information		
Sequencing method	PacBio	Illumina
Total bases (bp)	659,984,259	3,107,325,716
Total reads	69,490	20,578,316
N50 (bp)	11,164	
GC content	43.45%	43.64%
AT content	56.55%	56.36%
SRA accession no.	SRR27792686	SRR27792687

Genomic information	
Genomic element	Chromosome
Size (bp)	5,369,373
GC content	43.5%
Number of coding sequences	4,371
Number of rRNAs	18
Number of tRNAs	74
Genbank accession no.	CP098482

**Fig 1:**
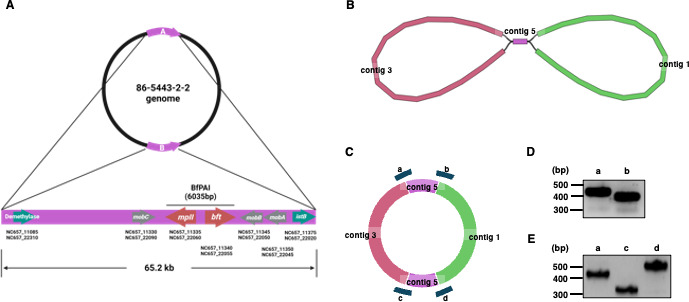
The genome structure of ETBF strain 86-5443-2-2 with two *B. fragilis* pathogenicity island (BfPAI) fragments. (**A**) The two fragments are positioned opposite each other. We indicate some genes located in the fragment. The green arrows are the first and the last gene of the fragment except hypothetical protein. The gray arrows are the mobilization genes located on both sides of BfPAI. Red arrows are the toxin genes, *bft* and *mpII*, located on BfPAI. (**B**) Diagram of the draft for ETBF strain 86-5443-2-2 genome resulting from FLYE v2.6 program. There are three contigs, two contigs (contigs 1 and 3) and one node (contig 5). (**C**) Diagram of the potential genome structure with two fragments. Target regions of polymerase chain reaction (PCR) to verify the genome structure (**A–D**). (**D–E**) Agarose gel electrophoresis of PCR products was obtained using specific primers for the contig 5 from panel C (**A–D**). PCR was performed with an initial denaturation at 95°C for 2 minutes, followed by denaturation cycles at 95°C for 30 seconds, annealing cycles at 65°C for 1 minute, extension cycles at 72°C for 10 minutes, and a final extension at 72°C for 5 minutes during 25 cycles. The PCR reaction mixture composition included colony of ETBF strain 86-5443-2-2, 25 uL Pfu Master Mix (BioFACT, cat#PD301-50h), 2 uL forward primer (5 pmole/uL), 2 uL reverse primer (5 pmole/uL), and 21 uL DEPC water.

Using default software parameters for genome assembly, we initially created a draft using long reads (NCBI SRR27792686) from PacBio Sequel with FLYE v.2.6 (4) (Fig. 1B). The draft wasn't circular, leading to our hypothesis of a single node between contig 1 and contig 3 (Fig. 1B). To verify this, we amplified the fragment using PCR primers (Fig. 1D and E) and then sequenced it to confirm the sequences between contigs (https://doi.org/10.6084/m9.figshare.28140293.v1). After PCR confirmation, we obtained the final genome draft (Fig. 1C). To enhance the sequence’s accuracy, a polishing process was undertaken, incorporating short reads (NCBI SRR27792687) from the Illumina platform using Polypolish v0.5.0 (5), with the final genome draft (Fig. 1C) serving as the template for these long-read sequences. The comprehensive genome is 5,369,373 base pairs long with 43.5% GC content. Annotation using the NCBI Prokaryotic Genome Annotation Pipeline (PGAP, version 6.4) (6, 7) revealed 4,465 genes and 4,310 proteins. The complete sequencing data is available in GenBank (CP098482).

Notably, the ETBF strain 86-5443-2-2’s genome contains two identical 65.2 kb fragments (Fig. 1A), initially thought to be a single node (Fig. 1B). PCR confirmed both copies of contig 5, positioned in opposite directions (Fig. 1C through E) using a forward primer from contig 1 or 3 and a reverse primer from contig 5. These fragments house the *B. fragilis* pathogenicity island (BfPAI) (8), featuring toxin genes *bft* and *mpII* (Fig. 1A), possibly related to increased virulence in ETBF strain 86-5443-2-2 (9).

## Data Availability

The data that support the findings of this study are openly available in GenBank of NCBI under the accession no. CP098482 for complete genome sequencing, SRR27792686 for the PacBio sequencing data, and SRR27792687 for the Illumina sequencing data is deposited in NCBI database. PCR primers and sequences of the PCR fragment have been deposited in Figshare (https://doi.org/10.6084/m9.figshare.28140293.v1).
